# Pediatric Psoriasis: From New Insights into Pathogenesis to Updates on Treatment

**DOI:** 10.3390/biomedicines9080940

**Published:** 2021-08-02

**Authors:** Hye One Kim, Seok Young Kang, Jin Cheol Kim, Chun Wook Park, Bo Young Chung

**Affiliations:** 1 Department of Dermatology, Hallym University Kangnam Sacred Heart Hospital, Seoul 07441, Korea; hyeonekim@gmail.com (H.O.K.); tjdjrdud@naver.com (S.Y.K.); aiekfne@naver.com (J.C.K.); dermap@hanmail.net (C.W.P.); 2 Department of Dermatology, Hallym University College of Medicine, Chuncheon 24252, Korea

**Keywords:** psoriasis, pediatric, childhood, pathogenesis, topical treatment, systemic treatment, biologics

## Abstract

Psoriasis is a chronic inflammatory systemic disease primarily affecting the skin, but which often involves considerable comorbidities as well. One-third of psoriasis cases start during childhood. In pediatric psoriasis, an association with several medical comorbidities is also indicated. Furthermore, because of its chronic nature and frequent relapses, psoriatic patients tend to require long-term treatment and experience negative impacts on their quality of life. Considering the different clinical characteristics of pediatric psoriasis, it has recently been presented that the pathogenesis of pediatric psoriasis is distinct from adult psoriasis. Treatment for pediatric psoriasis usually involves the same methods as for adults. However, most treatments in pediatric psoriasis are used off-label and research in this regard is still lacking. Targeted therapies involving newly developed biologics are also increasingly being applied to psoriasis in children. This review summarizes the clinical characteristics of pediatric psoriasis and focuses mainly on the updated concepts of pathogenesis and treatments in pediatric psoriasis. This was undertaken to widen the understanding of these relevant aspects and to provide better management of pediatric psoriasis by clinicians.

## 1. Introduction

Psoriasis is a chronic immune-mediated inflammatory disease that mostly affects the skin, nails, and joints. Psoriasis in children is relatively common, affecting approximately 2% of the global population, and approximately one-third of psoriasis cases are present during childhood. According to most studies, girls have a slightly higher prevalence of pediatric psoriasis than boys [1,2]. Psoriasis, as a systemic disease, can be associated with significant comorbidities, and its relation with such comorbidities is also indicated in the pediatric population. According to previous research, the prevalence of comorbidities in children (i.e., patients younger than 20) with psoriasis (14.4%) is nearly two-times higher than that in children without psoriasis (7.2%) [3]. Pediatric psoriasis patients have a two-to-four–fold higher risk of hyperlipidemia, hypertension, diabetes mellitus, rheumatoid arthritis, and Crohn’s disease [4,5,6]. Psoriatic arthritis is a well-documented comorbidity in children, with a confirmed prevalence ranging from 6% to 41% in those with psoriasis [7,8]. Detecting this disease early in life and applying appropriate treatment may delay or even prevent serious impacts on a child’s quality of life and comorbidities. Therefore, having an accurate understanding the various aspects of this disease in this age group is paramount.

## 2. Clinical Presentation of Pediatric Psoriasis

Psoriasis has a wide range of symptoms and degrees of severity. The overall clinical manifestation of psoriasis in children is similar to that of adult psoriasis. However, some clinical characteristics of pediatric psoriasis are notable, including a predilection for involvement of the face and anogenital areas, a higher prevalence of neonatal diaper rash and guttate psoriasis, and smaller and softer plaques than in adults [1,9].

Anogenital psoriasis is the most common form of psoriasis in children under the age of two. Children are more likely than adults to experience spontaneous remission, which may be related to severity and triggering factors [10,11].

A subset of children’s plaque psoriasis can be smaller, thinner, and less scaly than adults. Psoriasis in infants often affects the diaper region, resulting in a wide area of confluent erythema or salmon-colored patches or plaques [12,13]. Scaling cannot be detected due to the moisture from occlusion of the diaper region, making it difficult to distinguish diaper psoriasis from other common diaper rashes. The presence of umbilical involvement or the spread of traditional plaques may help differentiate it from other causes of diaper rash [3,14,15].

Guttate psoriasis appears as several small, erythematous, droplet like, scaly papules on the trunk and extremities, and is more common in children (13.7%). Infections, especially group A beta hemolytic streptococcus, often precede the onset or flare of guttate psoriasis. Interestingly, recent research has found that patients with guttate psoriasis associated with streptococcus infection usually achieved spontaneous remission, while those without prior signs of infection had continued symptoms or even worsened to plaque psoriasis [12,13,16].

## 3. Etiopathogenesis of Pediatric Psoriasis

Many non-specific factors can trigger psoriasis in children, such as high stress when reaching the age to start school, detachment from parents, mild mechanical trauma (Koebner phenomenon), and infection was reported in a prior study [17]. However, systemic drugs (lithium, beta blockers, antimalarial agents, etc.) or HIV infection, are less frequently implicated in children than in adults [1].

**Figure 1:**
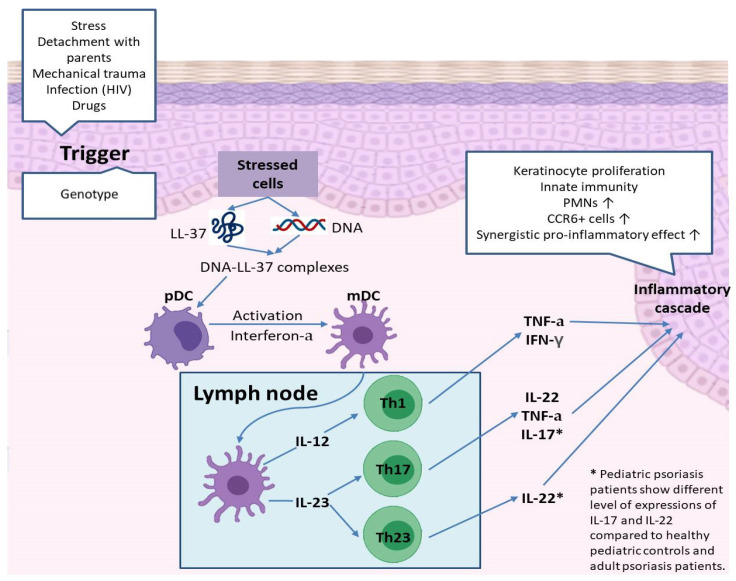
Pathogenesis of psoriasis focusing on the immunologic aspect.

Pediatric psoriasis patients exhibited a distinct difference in the expression of interleukin 17 (IL-17) and IL-22 compared to that of healthy pediatric controls and adult psoriasis patients. Figure 1 depicts a schematic drawing showing the pathophysiology of psoriasis. Following triggering events, stressed cells release LL-37 and DNA, forming DNA-LL-37 complexes. Activated myeloid dendritic cells secrete IL-12 and IL-23, which play central roles in the differentiation of naïve T cells to T helper (Th) cells. IL-12 induces differentiation of Th1 and IL-23 to Th17 and Th23. These differentiated T helper cells secrete various cytokines, which in turn promote an inflammatory cascade.

The immunopathogenesis of psoriasis has been investigated in many recent studies [18,19]. These studies suggest, in common, that interactive reaction between immune cells in the skin, such as keratinocytes, T helper cell 1 (Th1), Th17, Th22, neutrophils, and mast cells, along with the abnormal expression of cytokines such as interferon alpha (IFN-a), tumor necrosis factor alpha (TNF-a), IL-17, IL-22, and IL-23; are mainly implicated in the occurrence of psoriasis [20,21]. Moreover, the imbalance between effector and regulatory T cells (especially Th-17) has been proposed as a key mechanism of psoriasis [22]. Genetic factors are also largely involved in the pathogenesis of psoriasis. It has been elucidated that multiple alleles (HLA-Cw6, HLADQ*02:01, CCHCR1, and CYP1A1) and loci (PSORS1-9, PSORSASI) are implicated in the development of psoriasis [23,24]. One European study showed that the risk of psoriasis is nearly 40% if both parents are affected, 14% if one parent is affected, and 6% if a sibling is affected [25].

Psoriasis in childhood is different from adult psoriasis in its clinical manifestation, from which it can be inferred that different pathogenic processes may be involved [26,27,28]. However, the different pathogenic process in pediatric psoriasis has not well elucidated. Compared to adult psoriasis, wherein immunologic and molecular data have been investigated to develop novel treatment options, there have been relatively few studies on psoriasis in childhood. A recent study revealed that the expressions of TNF-α and IL-17 in the skin lesions of pediatric psoriasis patients have been shown to be different from those of adult psoriasis patients [26]. In another study, significant differences in IL-17 and IL-22 expression were observed in pediatric psoriasis patients, relative to those in healthy pediatric controls and adult psoriasis patients. Lesional tissue from pediatric psoriasis patients is associated with higher levels of IL-22-producing T cells and relatively fewer IL-17-producing T cells compared with adult psoriasis [27]. Zhang et al. reported that increased Th17 and regulatory T cells in the peripheral circulation were positively correlated with the disease severity [28]. Thus, the distinct immunophenotypic findings in pediatric psoriasis may be helpful to determine targeted therapies for pediatric psoriasis. However, previous relevant studies were small pilot studies with small sample size. In this regard, more and larger-scale studies are warranted to investigate these specific immunophenotypic targets for pediatric psoriasis.

## 4. Treatments of Pediatric Psoriasis

Most of the medications approved for adult psoriasis can be used in children as well, although it is required that an off-label prescription is needed in pediatric cases. Not only efficacy and safety studies, but the outcomes from long-term follow-up are insufficient in this population [29,30]. 

### 4.1. Topical Treatments

The primary topical treatment for all subtypes of psoriasis includes corticosteroids, calcineurin inhibitors, vitamin D3 analogs, and keratolytics [31]. Among these treatments, corticosteroids have been the most commonly used, due to their anti-inflammatory and anti-proliferative effects. Vitamin D3 analogs, including calcipotriol and calcitriol, can inhibit keratinocyte proliferation and induce their differentiation. Such analogs can be used as a monotherapy or in combination therapy with corticosteroids, anticipating a synergistic effect. Although safety and efficacy in children are rarely reported, topical treatments can be used with relatively lower risk of adverse effects than with systemic medications [32].

#### 4.1.1. Emollients and Keratolytics 

Emollients and keratolytics have an additive role after the primary treatment of psoriasis, such as with topical or systemic medications, or phototherapy [33]. Keratolytic agents (urea, salicylic acid) are used for hyperkeratotic lesions, whereas emollients are suitable for common plaque-type scaly lesions [33]. Emollients can also be used in the remission phases of the disease. In infants, emollients can be the only treatment in the absence of pruritus, notably for napkin psoriasis and guttate psoriasis [34]. Topical salicylic acid should be used with caution in children because even if a small amount of salicylic acid is applied, the amount absorbed into systemic circulation may be high, and accordingly, it may cause central nervous system and renal side effects. This agent should be avoided in infants, and can be used over a small area with concentration up to 0.5% in those of preschool age [35,36].

#### 4.1.2. Topical Corticosteroids

Topical corticosteroids are commonly used as a first-line treatment for pediatric psoriasis with localized disease [30,37]. The selection of topical preparations should take into account a number of factors, including the area and extent of the lesion, the type and thickness of the psoriasis skin lesion, and the patient’s age [30]. Recently, two studies elucidated the efficacy and adverse effects of high-potency topical corticosteroids: halobetasol cream 0.05% and clobetasol propionate emulsion 0.05%. These two studies showed efficacy after 2 weeks of treatment and local side effects including hypopigmentation and skin atrophy [38]. Herz et al. presented the efficacy of halobetasol monotherapy in 11 pediatric psoriasis patients for 2 weeks. Among them, eight had complete remission by the end of the study [38]. A report by Frangos and Kimball revealed the efficacy of clobetasol foam after 2 weeks of treatment in child patients with psoriasis [38]. However, in patients under 12 years of age, reversible inhibition of the HPA axis was observed. Due to the risks of these side effects, high-potency steroids should be used only for a short period of time. Meanwhile, low-potency topical corticosteroids can be applied on the thin skin areas such as face and genital regions with a low risk of side effects [39]. 

Although the use of topical corticosteroids has good results in the pediatric population, they should be applied with caution due to adverse effects. In particular, infants, toddlers, and young children (aged 0 to 6 years) are more susceptible to HPA suppression because their body surface area is large compared to their body volume. In the case of guardians, it is noted that if a high-potency topical steroid is used and then abruptly discontinued, a rebound flare may occur [40,41]. A topical high-potency steroid may be used for 1 week, then exchanged for moderate-to-mild–potency steroids, and application to folds, the face, and the genital area should be avoided.

#### 4.1.3. Topical Calcineurin Inhibitors (TCI)

Topical calcineurin inhibitors include tacrolimus (0.03% ointment) and pimecrolimus (1% cream), which is approved for children with atopic dermatitis [42]. By inhibiting the activity of calcineurin, TCI diminishes the formation of IL-2 and subsequently reduces T-cell activity and proliferation. Several studies have shown the efficacy of TCI treatment for psoriasis in the pediatric population [40,42,43,44]. According to one systematic review about pediatric psoriasis, TCI is most commonly used to reduce the use of corticosteroids in sensitive areas, such as the face, genitalia, and intertriginous areas [45]. In one study, eleven patients (6–15 years old) with psoriasis were treated with tacrolimus 0.1% to the face and fold areas for 6 months. Of these patients, 12% achieved complete remission, and most of the other patients reported improvement of more than 90% [46]. Another study showed that the daily application of tacrolimus 0.1% ointment to treat pediatric inverse psoriasis patients (22 months to 16 years old) resulted in complete clearing in all patients within 2 weeks [43]. In two case reports, topical pimecrolimus was also applied successfully in infants and children with psoriasis on the face and genital area. In another study, pediatric patients with periocular and anogenital psoriasis showed remission after using pimecrolimus for 20 days [44].

Burning and stinging sensations are frequently reported as side effects of TCI use in children [42]. In 2006, the US FDA issued a boxed warning that chronic application of TCI might enhance the theoretical risk of lymphoma. However, evidence that long-term use of topical tacrolimus or pimecrolimus increases the risk of lymphoma does not exist to date [47].

#### 4.1.4. Topical Vitamin D Analogues

There are several studies showing that vitamin D analogs can be used safely and effectively in pediatric psoriasis [37,43]. The vitamin D analogs (including calcipotriene, calcipotriol, and calcitriol) have been known to inhibit proliferation and promote differentiation of keratinocytes, which warrants a synergistic effect with corticosteroids [37]. In one randomized double-blind study, 77 children (2–14 years old) were treated with calcipotriol for all body lesions except for the face, scalp, and genital area during 8 weeks [48]. A reduction of 52% in the Psoriasis Area and Severity Index (PASI) score was observed in the calcipotriol group (43 patients) and a 37.1% reduction in the placebo group (34 patients) [48]. A systematic review of the literature conducted by Jager and his colleagues suggested that vitamin D-analog monotherapy could be considered as the first line treatment for pediatric psoriasis [49]. However, this is not the common approach for the treatment of pediatric psoriasis in clinical settings.

Vitamin D3 derivatives usually have the side effect of an irritating sensation and should be avoided on the face, genitalia, and intertriginous areas [50]. These derivatives are often used in conjunction with emollients to improve these side effects. Although there are no recommendations and data available on the maximum dose for children, caution should be exercised with the dosage as there may be theoretical risks, such as hypercalcemia and hypovitaminosis D, due to systemic absorption, especially if psoriasis is widespread, in young infants, and with widespread occlusion therapy [48]. Vitamin D derivatives are commercially available as a single formulation or as a combination of betamethasone dipropionate in a cream, ointment, suspension, or foam formulation. As the maximum usable dose to prevent hypercalcemia in adolescents, a limiting criterion of 80 g/week for scalp composites is suggested, but in the case of pediatric patients, there are no clear guidelines yet. Vitamin D derivatives must be used carefully in the presence of calcium metabolism disorder or kidney disease at any age [51].

The combined topical therapies with vitamin D3 derivatives and other topical agents have been used for the treatment of pediatric psoriasis. For children older than 12 years of age, the calcipotriol/betamethasone dipropionate compounded agents have been approved by the FDA for use on the body and scalp. In a Phase 2 open-label trial, 31 pediatric scalp psoriasis patients (12–17 years old) were treated with a compounded suspension of calcipotriol and betamethasone dipropionate once daily for 8 weeks. Remission was found in eight patients (58%) [52]. Although 65% of patients showed itching at the beginning of treatment, their conditions mostly improved, with the itching rate reduced to 10% by the end of the study [52]. Preapplication of 6–10% salicylic acid 1 week prior to single treatment with calcipotriene may also be beneficial. Using the emollient with calcipotriol at the same time, or separately, is a way to increase the effect while reducing side effects such as stinging, burning, and itching [52].

#### 4.1.5. Other Topical Agents 

Tazarotene, a third-generation topical retinoid, is approved by the FDA for the treatment of adult plaque psoriasis. However, there are no reported studies focused on tazarotene in the pediatric and adolescent groups. There was only a case report of successful results for a 6 yearold nail psoriasis patient treated with 0.05% tazarotene gel daily for 8 weeks. As tazarotene may cause birth defects, prescription to adolescent girls of possible childbearing age should applied with caution [53].

Anthralin (dithranol) has effects on reducing inflammation and proliferation by inhibiting DNA synthesis, and can be used in children safely [54]. In one study, a retrospective chart review was conducted on 60 psoriasis patients (3–18 years old) treated with anthralin [55]. Although the treatment had an excellent effect in only 3.7% of the patients, almost 70% of the patients presented a good response. In another retrospective study, 0.1–2% dithranol cream was applied to 58 pediatric psoriasis patients for 30 min daily [55], and 81% of patients achieved complete remission of skin lesions within 2 months after treatment. A prospective study of 34 pediatric psoriasis patients with failed topical steroid and calcipotriene treatment was conducted. The average treatment period was 11 weeks. The Children’s Dermatology Life Quality Index (CDLQI) showed an improvement of 5.1 points and PASI was also reduced by 69% [56]. Anthralin has side effects, such as irritation and discoloring of the skin.

Topical coal tar also acts as an anti-proliferative and anti-inflammatory agent in psoriasis [57]. Although the exact mechanism of action has not yet been identified, it is considered to act as an aryl hydrocarbon receptor agonist [58]. It can be used as a single agent but there are no studies where it has been applied to treat pediatric psoriasis as a single agent. In a retrospective study of 54 children with plaque or guttate psoriasis aged 1–16 years, the treatment effect of coal tar and phototherapy was analyzed [57]. As a result, 64% of the patients showed remission of their lesions. Of these patients, 83% maintained remission after 4 months, but only 43% maintained remission after 12 months. In a retrospective study of 65 patients (3 months to 18 years) with recurrent psoriasis, coal tar with phototherapy presented a notable improvement in all patients, of which 85% experienced sustained remission [58]. However, continuous use can increase the risk of cancer, so other alternative treatment options can be considered as a way to reduce this risk [59].

Tapinarof is an aryl hydrocarbon receptor (AhR) modulating agent used for the treatment of psoriasis [60]. The efficacy of tapinarof for psoriasis is attributed to its specific binding and activation of AhR, a ligand-dependent transcription factor, leading to the downregulation of proinflammatory cytokines, including interleukin 17, and to the regulation of skin barrier protein expression to promote skin barrier normalization with antioxidant activity [61,62]. A randomized, double-blind, vehicle-controlled Phase 3 study is in an ongoing clinical trial (NCT03983980) to evaluate the efficacy and safety of topical tapinarof 1% cream used once daily for the treatment of plaque psoriasis in adults [63]. In a Phase 2b, double-blind, vehicle-controlled study, tapinarof was efficacious and well tolerated in adolescents with atopic dermatitis [63]. However, no study on pediatric psoriasis has been conducted yet. Further investigations of this agent are warranted in these age groups.

### 4.2. Phototherapy

Phototherapy is frequently considered in clinical situations of childhood patients with refractory plaque or guttate psoriasis, which involve diffuse areas of the body, in cases of severe palmoplantar disease, or in patients who cannot tolerate systemic medications [64,65]. Several case series published in the last five years have shown the effectiveness of narrow band ultraviolet-B (NB-UVB) phototherapy in children. NB-UVB (311–313nm) has been found to be reliable and safe in the treatment of pediatric plaque and guttate psoriasis. A retrospective analysis of the effect of NB-UVB phototherapy in pediatric psoriasis patients aged 5–17 years found that 92% of the patients improved significantly with almost no side effects [66]. Another similar retrospective study revealed that, after treatment with NB-UVB, pediatric psoriasis patients showed satisfactory improvement without side effects in over 60% of patients [67]. A prospective study of 20 patients (aged 6–14 years) with recurrent guttate or plaque pediatric psoriasis showed that when NB-UVB was used twice a week, the result was that a minimum of 60% of patients were improved at 12 weeks of treatment. In this study, a remission rate of almost 90% was observed [67].

For the treatment of psoriasis in children, excimer laser, ultraviolet-A (UVA) light with, psoralen (topical or oral) may be effective and well-tolerated [68], but the evidence is relatively insufficient [51]. Psoralen ultraviolet-A (PUVA) is particularly associated with the development of lentigines, folliculitis, polymorphic light eruption, and onycholysis. In particular, it can cause skin cancer when used for a long time. Psoralen should be avoided in pediatric patients less than 12 years of age [51].

In most patients, it takes more than 4 weeks after the initial treatment for the treatment effect to appear, and during this process, the patient’s compliance is often poor. Allowing children to feel familiar with the equipment and lights, as well as including the family in the care decisions, improves the compliance of children with the treatments [51]. 

### 4.3. Systemic Treatments 

Systemic agents including methotrexate (MTX), cyclosporine, and acitretin are used for the treatment of pediatric psoriasis (Table 1), based on the accumulated knowledge of their advantages and risks in children with other disorders such as ichthyosis, juvenile rheumatoid arthritis, and organ transplantation, respectively. To optimize their advantages while minimizing side effects, these agents may be combined with topical drugs or phototherapy, or used in a sequential approach [51].

The subtype of psoriasis, the rate of disease progression, comorbidities, and unsuccessful topical and phototherapies are all factors that should be considered when determining whether to use systemic treatment in pediatric psoriasis patients. Considering the special characteristics of the metabolism of children, maintaining long-term remission at the lowest possible dose is preferred [1,15].

#### 4.3.1. Methotrexate 

MTX is a systemic immunosuppressant that is most widely used in the care of moderate-to-severe pediatric psoriasis patients and has long-term effects with safety evidence [69]. MTX is a folic acid antagonist that binds to and inhibits the dihydrofolate reductase in an irreversible manner, inhibiting RNA and DNA synthesis, and causing cell-cycle arrest. It also has an anti-inflammatory effect which is mediated via the adenosine pathways [70]. MTX is approved for adult psoriasis as well as for inflammatory bowel disease (IBD) and juvenile arthritis in children [71].

In a retrospective study, 24 patients with severe recurrent pediatric psoriasis were given a weekly dosage of 7.5 to 20 mg, and 22 of them achieved a PASI of 75. The majority of patients showed a response within 5 weeks. Nine patients had minor stomach side effects (nausea, vomiting), but not enough to cause them to avoid taking the drug [72]. In 24 pediatric psoriasis patients, a prospective long-term retrospective analysis about the impact of MTX treatment on patient quality of life was performed [72]. MTX was given to the patients once a week at a dosage of 0.14 to 0.63 mg/kg. The average CDLQI score decreased from 9.0 to 3.8 and the PGA score decreased from 3.0 to 1.2 after 24 weeks of follow-up [72]. A cohort study of 289 pediatric psoriasis patients was performed, with the participants being children 9–14 years old who were treated with MTX, acitretin, and cyclosporine [73]. Most of the patients (90.8%) treated with MTX reported no side effects. Eight percent of patients showed nausea, and one patient stopped treatment because of an abnormal level of liver enzymes. No medication was superior to another, with 34.1% of MTX patients achieving a PASI of 75 or higher [73]. 

The recommended dosage of MTX in children under the age of 13 should be adjusted based on body weight, beginning at 0.2 to 0.3 mg/kg/week, then titration to 1.25 to 5 mg/week, raising the dose until it is successful. For pediatric patients over 13 years of age, the dose is regulated similarly to adults [56]. 

In general, the treatment effect improves 5 to 12 weeks after beginning treatment, but it may take 3 to 4 months to achieve the full treatment effect in pediatric patients. There is no established consensus for the duration of treatment, but slow tapering 2–3 months after remission is a reasonable treatment protocol. MTX treatment requires daily supplementation with 1 mg folic acid [74].

Among side effects, elevated liver enzymes are the most common, and it is recommended that the liver function test should be checked, at least 4–6 days after treatment. Bone marrow suppression normally happens during the first 4–6 weeks of therapy, and it is also recommended to conduct a complete blood count 5 to 6 days after the initial administration. As taking drugs that block the metabolic pathways of folic acid, or taking NSAIDs that reduce renal clearance may increase the risk of bone marrow toxicity, these drugs with MTX should be used cautiously [51]. MTX should be avoided during pregnancy or if the patient has liver disease, and it should be used with caution in children who have poorly controlled diabetes or obesity [51]. 

#### 4.3.2. Cyclosporine

Cyclosporine is an immunosuppressant drug that has been approved for the treatment of generalized pustular psoriasis and severe recalcitrant psoriasis in adults. Cyclosporine inhibits T lymphocytes and prevents the development of IL-2 and IFN-gamma, thereby blocking the signal pathway linked to psoriasis pathogenesis [75]. It can be used in severe plaque, pustular, and erythrodermic psoriasis in children with rapid efficacy. One report focused on the effects of cyclosporine on a 10-month-old boy with generalized pustular psoriasis at a dosage of 1 mg/kg/d [76]. The patient’s condition changed within four weeks of starting the treatment, and after six months, the drug was discontinued. After 2 years of follow-up, the disease was maintained in a remission state [77]. The effect of cyclosporine was studied in 22 pediatric psoriasis patients who were resistant to other treatments [78]. Seventeen (77%) of the patients had a good response and achieved remission within 4 weeks. As a result of analyzing 10 pediatric psoriasis patients in a retrospective study, seven (70%) of the patients achieved PASI 75 or higher within an average of 4 weeks [76]. Two of the patients complained of stomach pain and had high creatinine levels [76].

Nephrotoxicity is one of the side effects of most concern. The mechanism of cyclosporin nephrotoxicity suggests activation of the intrarenal renin angiotensin system and associated vasoconstriction. Chronic nephrotoxicity leads to histological changes featuring obliterative vasculopathy and tubulointerstitial fibrosis [79]. As high blood pressure can develop as a first sign of nephrotoxicity, it is important to monitor blood pressure during treatment. Cyclosporine is not recommended for patients who have had or are now receiving PUVA treatment because long-term use has been linked to the development of non-melanoma skin cancer [51].

Other cyclosporine-related side effects include gastrointestinal upset, hypertrichosis, arthralgia, and gingival hyperplasia. Although, as a side effect of cyclosporin, lymphoproliferative malignancies were reported in pediatric organ transplant patients, the risk is also very low at the doses and durations used in the treatment of psoriasis [51]. 

#### 4.3.3. Systemic Retinoids

Retinoids are vitamin A analogs that modify cellular metabolic pathways, cellular differentiation, and apoptosis through binding to retinoic acid receptors [79,80]. Psoriasis is treated with systemic retinoids, which reduce inflammation and control keratinocyte maturation. In addition, they have the function of inhibiting neutrophil chemotaxis, which is particularly effective in pustular psoriasis. 

Acitretin, a second-generation synthetic retinoid, is the pharmacologically active metabolite of etretinate [81]. Acitretin is particularly effective as a maintenance treatment for widespread guttate psoriasis, and palmoplantar psoriasis [82]. One study focused on the effects of acitretin in 17 patients with generalized or pustular psoriasis who were between the ages of one month and 13 years old [82,83]. Of the patients who received systemic acitretin monotherapy, 14 patients (82%) showed remission, and 3 patients showed remission after receiving cyclosporine combination treatment. No additional side effects were observed. In a retrospective medical chart review of 15 children with generalized or palmoplantar psoriasis, as a result of studying the therapeutic effect of acitretin monotherapy, 14 out of 15 patients (93%) showed disease remission without side effects [83].

Acitretin is available in 10 mg, 17.5 mg, and 25 mg capsules and is commonly administered in doses of 0.1 to 1 mg/kg/d for children of all ages [51]. Dryness of the skin or mucous membranes is a common side effect of systemic retinoid formulations. Bone changes, such as hyperostosis, are a concerning complication of the long-term use of systemic retinoid in children. Due to the teratogenicity of oral retinoids, for adolescent girls treated with retinoids, education on pregnancy prevention and regular pregnancy monitoring should be considered. Isotretinoin can be used the substitute of acitretin in female adolescents due to its shorter clearance duration compared to acitretin [84,85]. 

#### 4.3.4. Systemic Fumaric Acid Esters

Fumaric acid esters are small molecules that have a variety of immunomodulatory effects, but their exact mechanism is unknown [74]. The use of dimethyl fumarate for adults with moderate-to-severe plaque psoriasis was approved by the European Medicines Association (EMA) in 2017 [86]. In the United States, fumaric acid esters are not officially approved for the treatment of psoriasis [56,71].

In one study, six pediatric patients were treated with fumaric acid esters and all patients had a substantial decrease in PASI and BSA after 12 weeks of treatment: 50% of them attained PASI 100 [87]. A multicenter retrospective chart review was conducted on 19 pediatric patients treated with fumaric acid esters [69]. Among them, 13 (68%) of the patients discontinued the drug treatment due to side effects such as abdominal pain, diarrhea, hot flashes, and headache. Two patients (10%) had significant side effects, including bone marrow suppression and pericarditis. Despite the short duration of therapy, fumaric acid esters had a higher rate of side effects than other systemic therapies [69].

#### 4.3.5. Other Systemic Agents

For the treatment of psoriasis in children, there is currently insufficient evidence on hydroxyurea, azathioprine, leflunomide, apremilast, mycophenolate mofetil, 6-thioguanine, and sulfasalazine. Only in specific cases can these be selected in consideration of individual clinical factors [69,88].

### 4.4. Biologic Therapy 

Biologics are treatments for serious and/or recalcitrant cases of plaque, pustular, and erythrodermic psoriasis, as well as those with concurrent psoriatic arthritis (Table 2). Several biologic agents have also recently been approved for use in pediatric psoriasis. When compared to other systemic therapies, biologics are very convenient because they have better dosage protocols and need less laboratory testing. Furthermore, because they are targeted drugs, the risk of systemic toxicity may be smaller. However, severe side effects have been identified in children with juvenile inflammatory arthritis who were treated with these drugs, including opportunistic infections, malignancies, latent tuberculosis, and autoimmune diseases. Before beginning treatment, all patients should be tested for tuberculosis and undergo laboratory tests [29]. 

Biologic medication is an immune modulator that regulates the inflammatory response by targeting specific pathways in cell signaling. Data on the safety and effectiveness of use in pediatric psoriasis patients are rapidly accumulating. The FDA did not approve any biologics for the treatment of pediatric psoriasis until 2009. However, as evidence has accumulated since then, etanercept and ustekinumab have been approved by the FDA for the treatment of psoriasis patients 4 years and older, and 12 years and older, respectively. Adalimumab is also approved for use in children aged 4 and up in Europe, along with etanercept and ustekinumab [89]. Other biologics targeting the IL-23/IL-17 axis or JAK inhibitors are often used off-label to treat pediatric psoriasis.

#### 4.4.1. Etanercept

Etanercept is a recombinant protein that blocks TNF- from binding to its receptor [90]. A case study on the use of etanercept for the treatment of moderate and recalcitrant psoriasis in far younger children (22 months) has been published [90]. In 2008, 211 patients with moderate to severe plaque psoriasis from the ages of 4 to 17 were treated for 12 weeks with either etanercept at a dosage of 0.8 mg/kg or placebo, followed by an open-label duration of 24 weeks and a second randomization at 36 weeks to examine the effects of withdrawal or treatment in a randomized, double-blind, Phase 3 clinical trial [91]. PASI 75 was seen in 57% of the children at the end of the first 12-week period, compared to 11% in the placebo group. PASI 75 was found in 61% of patients in an open-label extension trial, and PASI 90 was found in 30% of those who completed 96 weeks of therapy. In a study of 181 patients who received long-term etanercept, 145 (80.1%) patients showed adverse events, such as upper respiratory tract infections (24.9%), nasopharyngitis (17.1%), streptococcal pharyngitis (12.7%), headache (11.6%), and sinusitis (10.5%). During the follow-up period, there were no cases of opportunistic infection or malignant tumors [92,93].

#### 4.4.2. Adalimumab

Adalimumab is a fully human TNF- monoclonal antibody. In Europe, adalimumab has been approved for the treatment of psoriasis in children and adolescents (aged ≥4 years) who have had an insufficient reaction to topical therapy and phototherapy. This drug is not approved for pediatric psoriasis indication in the USA [94,95,96]. In 114 patients aged 4–17 years with serious plaque psoriasis, a randomized, double-blind, Phase 3 trial comparing the efficacy between adalimumab and MTX was performed [96]. The rate of achieving PASI 75 at the 16th week of treatment was found to be 58% for an adalimumab 0.8 mg/kg-full dose, 44% for 0.4 mg/kg-half dose, and 32% for oral MTX 0.1–0.4 mg/kg/week. The percentages of those that achieved clear or almost clear PGA scores were 61%, 41%, and 41%, respectively. Infection was one of the common side effects, but there was no significant difference among the three groups [94].

#### 4.4.3. Ustekinumab

Ustekinumab is a monoclonal antibody that targets the p40 subunit of IL-12 and IL-23. It is approved for the treatment of moderate-to-severe plaque psoriasis in adults in most countries [97]. Landells et al. divided 110 pediatric patients into three groups and treated one with a standard dose of ustekinumab, one with a half dose of ustekinumab, and no dose for the placebo group [97]. At 12 weeks, 80.6% of those with the standard dose, 78.4% with the half dose, and 10.8% of those in the placebo group achieved PASI 75, while 61.1% of those with the standard dose, 54.1% with the half dose, and 5.4% in the placebo group achieved PASI 90. The standard dose group achieved PGA 0/1 at a rate of 67.6%, the half dose group at 69.4%, and the placebo group at 5.4%. The three classes had equal rates of adverse events. The most serious side effect was an allergic reaction at the injection site. In a recent study comparing the effectiveness of ustekinumab with that of etanercept for treating pediatric psoriasis, ustekinumab was found to be more efficacious at 12 weeks than etanercept [69].

A retrospective case series was performed on 51 children (ages 7 to 18) with psoriasis who were treated with etanercept, adalimumab, and ustekinumab [98]. All three groups had a positive response (defined as a 5-month significant improvement in the PGA score). There were no significant side effects, with the most common symptoms being injection site pain (8.6%) and fatigue (7.5%) [98].

#### 4.4.4. Infliximab

Infliximab is a chimeric monoclonal antibody that binds to human TNF-α and prevents it from binding to its receptor in soluble and membrane-bound forms [99]. Many countries have approved it for use in adults with psoriasis and other diseases [99]. It has been approved by the FDA for use in children aged 6 years and older who have Crohn’s disease or ulcerative colitis [100]. However, it is not approved for the treatment of pediatric psoriasis in any country [100]. Infliximab had a consistently higher incidence of confirmed malignancies (66/100,000 for all malignancies and 44/100,000 for lymphomas) than background rates in the general pediatric population (16.8/1,000,000 for all malignancies and 2.4/100,000 for lymphomas), according to a newly published study [100]. Moreover, it has been reported that psoriasis can be caused in the pediatric population by treatment of IBD with infliximab [100].

#### 4.4.5. Secukinumab

Secukinumab is a fully humanized, monoclonal anti-IL-17A antibody. Pediatric patients (162 patients) received secukinumab with low dose or high dose, etanercept, or placebo for 12 weeks in a Phase 3 double-blind, randomized, controlled trial [101]. Both secukinumab doses showed superior efficacy compared to placebo with respect to the PASI 90 response (72.5% and 67.5% vs. 2.4%), PASI 75 response (80.0% and 77.5% vs. 14.6%) and IGA at week 12. The response tendency was sustained to week 52 (PASI 75/90/100: low dose secukinumab, 87.5%/75.0%/40.0% and high dose secukinumab, 87.5%/80.0%/47.5% vs. etanercept, 68.3%/51.2%/22.0% and IGA 0 or 1: low dose secukinumab, 72.5% and high dose secukinumab, 75.0% vs. etanercept, 56.1%). The safety profiles were similar to adult studies with secukinumab [101].

#### 4.4.6. Ixekizumab

Ixekizumab is a humanized monoclonal antibody that selectively binds to IL-17A. In a Phase 3 double-blind, randomized, placebo-controlled trial, patients aged 6 to <18 years with moderate to severe plaque psoriasis were randomized to receive ixekizumab or placebo through week 12 [102]. Ixekizumab was superior to placebo for PASI 75 (89% vs. placebo, 25%) at week 12. Ixekizumab also showed superior results in PASI 75 and PGA at week 4, improvement in quality of life, and complete skin clearance. The safety profile of ixekizumab was generally consistent with that in adults with moderate-to-severe plaque psoriasis [102].

#### 4.4.7. Guselkumab

Guselkumab is a monoclonal antibody against interleukin-23. In adult patients, Guselkumab demonstrated superior clinical responses and was better tolerated, compared with adalimumab [103]. However, no study on the efficacy and safety of the IL-23 antibody for pediatric patients has been published to date. A study is now being conducted (NCT03451851) to evaluate the efficacy and safety of guselkumab in relation to etanercept and placebo in pediatric patients with chronic plaque psoriasis [103]. 

#### 4.4.8. Risankizumab

Risankizumab is also an anti-IL-23 antibody. In adult patients, a Phase 2 48-week, multicenter, randomized, dose-ranging trial of risankizumab showed clinical responses superior to those associated with ustekinumab [104]. A study of subcutaneous risankizumab injection in relation to ustekinumab for pediatric participants with moderate-to-severe psoriasis is under way (NCT04435600) [104]. 

#### 4.4.9. Tofacitinib

Tofacitinib is in the Janus kinase (JAK) inhibitor class, and inhibits the enzymes JAK1 and JAK3. In studies conducted in adult patients, tofacitinib demonstrated efficacy non-inferior to etanercept and was superior to placebo, implying the possibility that tofacitinib might be considered an additional therapeutic option [105,106]. A recent open-label study of 47 pediatric psoriasis patients reported that tofacitinib showed significant improvement in efficacy and the quality of life. At week 12, 55.32% achieved PASI 75, with 70.21% at week 36. No severe side effects were noted [107].

## 5. Conclusions

Psoriasis in children poses a number of challenges: it has age-specific clinical characteristics, the appearance of which can change with time. Most treatment modalities for adult psoriasis have been used off-label for pediatric psoriasis. Although there have been limited studies, recent research proposed a difference in the expressions of TNF-α-IL-17-IL-22 in pediatric psoriasis patients compared to those of adult psoriasis patients. Biologics as targeted therapy are also gradually being used for pediatric psoriasis. It is reasonable to surmise that a more targeted treatment approach for pediatric psoriasis will be guided by the unique immunophenotypic characteristics of psoriasis in children. Further studies are needed to identify the different pathogenic processes in pediatric psoriasis. Though the narrative nature of this review is its main limitation, updated concepts of pathogenesis and treatments in pediatric psoriasis could broaden the understanding of unfamiliar concepts and help clinicians apply them to their patients in the clinic.

## Figures and Tables

**Table 1:** Systemic treatments for pediatric psoriasis.

Systemic Treatments	Mechanism	Dosage	Side Effects
Methotrexate	Inhibition of RNA and DNA synthesis Cell cycle arrest Inhibition of inflammatory cytokines such as tumor necrosis factor (TNF) and interleukin (IL-6, and 8)	0.2 to 0.7 mg/kg/week	Elevated liver enzymes Bone marrow suppression
Cyclosporine	Inhibition of T lymphocytes Prevention of the development of IL-2 and IFN-gamma	1 to 5 mg/kg/d	Possible nephrotoxicity in prolonged usage High blood pressure
Retinoids	Modification of cellular metabolic pathways, cellular differentiation, and apoptosis	0.1 to 1 mg/kg/d	Pruritus, cheilitis, hair loss, partial osteoporosis, elevated liver enzymes, hyperlipidemia, and paronychia
Fumaric acid esters	Small molecules with immunomodulatory effects	≤720 mg/day	Gastrointestinal complains, lymphocytopenia, eosinophilia, and flush

**Table 2:** Biologics for pediatric psoriasis.

Biologics	Mechanism	FDA/EMA Approval	Dosing	Safety
Etanercept	A fusion protein blocking TNF- from binding to its receptor	Approved in FDA for treatment of psoriasis in patients ≥4 years of age Approved in EMA for treatment of psoriasis in patients ≥6 years of age	SC injection of 0.8 mg/kg per week with a maximum of 50 mg per week	Upper respiratory tract infections, nasopharyngitis, streptococcal pharyngitis, sinusitis, headache, injection site reactions
Adalimumab	A fully human TNF- monoclonal antibody	Approved in EMA for treatment of psoriasis in patients ≥4 years of age	For patients 15 to 30 kg: SC injection of 20 mg at week 0 and then 20 mg every other week For patients >30 kg: SC injection of 40 mg at week 0 and then 40 mg every other week	Upper respiratory infections, uncomplicated infections, injection site reactions
Ustekinumab	A monoclonal antibody that targets the p40 subunit of IL-12 and IL-23	Approved in FDA/EMA for treatment of psoriasis in patients ≥12 years of age	For patients < 60 kg: SC injection of 0.75 mg/kg at week 0, week 4, and then every 12 weeks For patients 60 kg to 100 kg: SC injection of 45 mg at week 0, week 4, and then every 12 weeks For >100 kg: SC injection of 90 mg at week 0, week 4, and then every 12 weeks	Upper respiratory tract infection, headache, injection site reaction
Infliximab	Chimeric monoclonal antibody that binds to human TNF-α	Not approved for pediatric psoriasis in FDA/EMA	IV infusion of 3.3–5 mg/ kg at weeks 0, 2, and 6, then once every 7-8 weeks	Infusion reactions, upper respiratory tract infection, headache, rash, and cough

SC, subcutaneous, FDA, Food and Drug Administration, EMA, European Medicines Association.

## Data Availability

Not applicable.

## Abbreviations

IL	Interleukin
Th	T helper cell
INF-a	Interferon alpha
TNF-a	Tumor necrosis factor alpha
HPA	Hypothalamic–Pituitary–Adrenal
FDA	US Food and Drug Administration
PASI	Psoriasis Area and Severity Index
CDLQI	Children’s Dermatology Life Quality Index
AhR	Aryl hydrocarbon receptor
NB-UVB	Narrow band ultraviolet-B
UVA	Ultraviolet-A
MTX	Methotrexate
IBD	Inflammatory bowel disease
PGA	Physician global assessment
SC	Subcutaneous
EMA	European Medicines Association
JAK	Janus kinase

## References

[1] Bronckers I.M., Paller A.S., van Geel M.J., van de Kerkhof P.C., Seyger M.M. (2015). Psoriasis in Children and Adolescents: Diagnosis, Management and Comorbidities. Paediatr. Drugs.

[2] Tollefson M.M., Crowson C.S., McEvoy M.T., Maradit Kremers H. (2010). Incidence of psoriasis in children: A population-based study. J. Am. Acad. Dermatol..

[3] Parisi R., Symmons D.P., Griffiths C.E., Ashcroft D.M. (2013). Identification and Management of Psoriasis and Associated ComorbidiTy (IMPACT) project team. Global epidemiology of psoriasis: A systematic review of incidence and prevalence. J. Investig. Dermatol..

[4] Pietrzak A., Grywalska E., Walankiewicz M., Lotti T., Roliński J., Myśliński W., Chabros P., Piekarska-Myślińska D., Reich K. (2017). Psoriasis and metabolic syn-drome in children: Current data. Clin. Exp. Dermatol..

[5] Becker L., Tom W.L., Eshagh K., Benjamin L.T., Paller A.S. (2014). Excess adiposity preceding pediatric psoriasis. JAMA Dermatol..

[6] Jensen P., Zachariae C., Christensen R., Geiker N.R.W., Schaadt B.K., Stender S., Hansen P.R., Astrup A., Skov L. (2013). Effect of weight loss on the severity of psoriasis: A randomized clinical study. JAMA Dermatol..

[7] Liu J.T., Yeh H.M., Liu S.Y., Chen K.T. (2014). Psoriatic arthritis: Epidemiology, diagnosis, and treatment. World J. Orthop..

[8] Chiam L.Y., de Jager M.E., Giam Y.C., de Jong E.M., van de Kerkhof P.C., Seyger M.M. (2011). Juvenile psoriasis in European and Asian children: Similarities and differences. Br. J. Dermatol..

[9] Griffiths C.E., Barker J.N. (2007). Pathogenesis and clinical features of psoriasis. Lancet.

[10] Farber E.M., Nall M.L. (1974). The natural history of psoriasis in 5600 patients. Dermatologica.

[11] Romiti R., Maragno L., Arnone M., Takahashi M.D. (2009). Psoriasis in childhood and adolescence. An. Bras. Dermatol..

[12] Gudjonsson J.E., Thorarinsson A.M., Sigurgeirsson B., Kristinsson K.G., Valdimarsson H. (2003). Streptococcal throat in-fections and exacerbation of chronic plaque psoriasis: A prospective study. Br. J. Dermatol..

[13] Pérez-Lorenzo R., Zambrano-Zaragoza J.F., Saul A., Jiménez-Zamudio L., Reyes-Maldonado E., García-Latorre E. (1998). Autoantibodies to autologous skin in guttate and plaque forms of psoriasis and cross-reaction of skin antigens with streptococcal antigens. Int. J. Dermatol..

[14] Silverberg N.B. (2010). Update on pediatric psoriasis, part 1: Clinical features and demographics. Cutis.

[15] Silverberg N.B. (2015). Update on pediatric psoriasis. Cutis.

[16] Thorleifsdottir R.H., Sigurdardottir S.L., Sigurgeirsson B., Olafsson J.H., Petersen H., Sigurdsson M.I., Gudjonsson J.E., Johnston A., Valdimarsson H. (2016). HLA-Cw6 homozygosity in plaque psoriasis is associated with streptococcal throat infections and pronounced improvement after tonsillectomy: A prospective case series. J. Am. Acad. Dermatol..

[17] Ji Y.Z., Liu S.R. (2019). Koebner phenomenon leading to the formation of new psoriatic lesions: Evidences and mechanisms. Biosci. Rep..

[18] Chiricozzi A., Romanelli P., Volpe E., Borsellino G., Romanelli M. (2018). Scanning the Immunopathogenesis of Psoriasis. Int. J. Mol. Sci..

[19] Albanesi C., Madonna S., Gisondi P., Girolomoni G. (2018). The Interplay Between Keratinocytes and Immune Cells in the Pathogenesis of Psoriasis. Front. Immunol..

[20] Ogawa E., Sato Y., Minagawa A., Okuyama R. (2018). Pathogenesis of psoriasis and development of treatment. J. Dermatol..

[21] Baliwag J., Barnes D.H., Johnston A. (2015). Cytokines in psoriasis. Cytokine.

[22] Nussbaum L., Chen Y.L., Ogg G.S. (2021). Role of regulatory T cells in psoriasis pathogenesis and treatment. Br. J. Dermatol..

[23] Tsoi L.C., Stuart P.E., Tian C., Gudjonsson J.E., Das S., Zawistowski M., Ellinghaus E., Barker J.N., Chandran V., Dand N. (2017). Large scale meta-analysis characterizes genetic architecture for common psoriasis associated variants. Nat. Commun..

[24] Liu Y., Helms C., Liao W., Zaba L.C., Duan S., Gardner J., Wise C., Miner A., Malloy M.J., Pullinger C.R. (2008). A genome-wide association study of psoriasis and psoriatic arthritis identifies new disease loci. PLoS Genet..

[25] Andressen C., Henseler T. (1982). Erblichkeit der Psoriasis. Eine Analyse von 2035 Familienanamnesen [Inheritance of pso-riasis. Analysis of 2035 family histories]. Hautarzt.

[26] Kim J.C., Kim S.M., Soh B.W., Lee E.S. (2020). Comparison of Cytokine Expression in Paediatric and Adult Psoriatic Skin. Acta Derm. Venereol..

[27] Cordoro K.M., Hitraya-Low M., Taravati K., Sandoval P.M., Kim E., Sugarman J., Pauli M.L., Liao W., Rosenblum M.D. (2017). Skin-infiltrating, interleukin-22-producing T cells differentiate pediatric psoriasis from adult psoriasis. J. Am. Acad. Dermatol..

[28] Zhang L., Li Y., Yang X., Wei J., Zhou S., Zhao Z., Cheng J., Duan H., Jia T., Lei Q. (2016). Characterization of Th17 and FoxP3(+) Treg Cells in Paediatric Psoriasis Patients. Scand. J. Immunol..

[29] Cline A., Berg A., Bartos G.J., Strowd L.C., Feldman S.R. (2020). Biologic Treatment Options for Pediatric Psoriasis and Atopic Dermatitis-A Review. J. Clin. Aesthet. Dermatol..

[30] Haulrig M.B., Zachariae C., Skov L. (2021). Off-Label Treatments for Pediatric Psoriasis: Lessons for the Clinic. Psoriasis.

[31] Maul J.T., Anzengruber F., Conrad C., Cozzio A., Häusermann P., Jalili A., Kolios A.G.A., Laffitte E., Lapointe A.-K., Mainetti C. (2021). Topical Treatment of *Psoriasis Vulgaris*: The Swiss Treatment Pathway. Dermatology.

[32] Lamba S., Lebwohl M. (2001). Combination therapy with vitamin D analogues. Br. J. Dermatol..

[33] Jacobi A., Mayer A., Augustin M. (2015). Keratolytics and emollients and their role in the therapy of psoriasis: A systematic review. Dermatol. Ther..

[34] Thomas J., Parimalam K. (2016). Treating pediatric plaque psoriasis: Challenges and solutions. Pediatric Health Med. Ther..

[35] Silverberg N.B. (2010). Update on pediatric psoriasis, Part 2: Therapeutic management. Cutis.

[36] Fluhr J.W., Cavallotti C., Berardesca E. (2008). Emollients, moisturizers, and keratolytic agents in psoriasis. Clin. Dermatol..

[37] Shah K.N. (2013). Diagnosis and treatment of pediatric psoriasis: Current and future. Am. J. Clin. Dermatol..

[38] Kimball A.B., Gold M.H., Zib B., Davis M.W., Clobetasol Propionate Emulsion Formulation Foam Phase III Clinical Study Group (2008). Clobetasol propionate emulsion formulation foam 0.05%: Review of phase II open-label and phase III randomized controlled trials in steroid-responsive dermatoses in adults and adolescents. J. Am. Acad. Dermatol..

[39] Bhutani T., Kamangar F., Cordoro K.M. (2012). Management of pediatric psoriasis. Pediatr. Ann..

[40] de Jager M.E., de Jong E.M., van de Kerkhof P.C., Seyger M.M. (2010). Efficacy and safety of treatments for childhood psoriasis: A systematic literature review. J. Am. Acad. Dermatol..

[41] Herz G., Blum G., Yawalkar S. (1991). Halobetasol propionate cream by day and halobetasol propionate ointment at night for the treatment of pediatric patients with chronic, localized plaque psoriasis and atopic dermatitis. J. Am. Acad. Dermatol..

[42] Wang C., Lin A. (2014). Efficacy of topical calcineurin inhibitors in psoriasis. J. Cutan Med. Surg..

[43] Steele J.A., Choi C., Kwong P.C. (2005). Topical tacrolimus in the treatment of inverse psoriasis in children. J. Am. Acad. Dermatol..

[44] Brune A., Miller D.W., Lin P., Cotrim-Russi D., Paller A.S. (2007). Tacrolimus ointment is effective for psoriasis on the face and intertriginous areas in pediatric patients. Pediatr. Dermatol..

[45] Carr W.W. (2013). Topical calcineurin inhibitors for atopic dermatitis: Review and treatment recommendations. Paediatr. Drugs.

[46] Malecic N., Young H. (2016). Tacrolimus for the management of psoriasis: Clinical utility and place in therapy. Psoriasis.

[47] Mansouri P., Farshi S. (2006). Pimecrolimus 1 percent cream in the treatment of psoriasis in a child. Dermatol. Online J..

[48] Oranje A.P., Marcoux D., Svensson A., Prendiville J., Krafchik B., Toole J., Rosenthal D., de Waard-van der Spek F.B., Molin L., Axelsen M. (1997). Topical calcipotriol in childhood psoriasis. J. Am. Acad. Dermatol..

[49] van Geel M.J., Mul K., Oostveen A.M., van de Kerkhof P.C., de Jong E.M., Seyger M.M. (2014). Calcipotri-ol/betamethasone dipropionate ointment in mild-to-moderate paediatric psoriasis: Long-term daily clinical practice data in a prospective cohort. Br. J. Dermatol..

[50] Gooderham M., Debarre J.M., Keddy-Grant J., Xu Z., Kurvits M., Goodfield M. (2014). Safety and efficacy of calcipotriol plus betamethasone dipropionate gel in the treatment of scalp psoriasis in adolescents 12–17 years of age. Br. J. Dermatol..

[51] Menter A., Cordoro K.M., Davis D., Kroshinsky D., Paller A.S., Armstrong A.W., Connor C., Elewski B.E., Gelfand J.M., Gordon K.B. (2020). Joint American Academy of Dermatology-National Psoriasis Foundation guidelines of care for the management and treatment of psoriasis in pediatric patients. J. Am. Acad. Dermatol..

[52] Seyger M., Abramovits W., Liljedahl M., Hoejen M.N., Teng J. (2020). Safety and efficacy of fixed-dose combination cal-cipotriol (50 μg/g) and betamethasone dipropionate (0.5 mg/g) cutaneous foam in adolescent patients (aged 12 to <17 years) with plaque psoriasis: Results of a phase II, open-label trial. J. Eur. Acad. Dermatol. Venereol..

[53] Diluvio L., Campione E., Paternò E.J., Mordenti C., El Hachem M., Chimenti S. (2007). Childhood nail psoriasis: A useful treatment with tazarotene 0.05%. Pediatr. Dermatol..

[54] de Jager M.E., van de Kerkhof P.C., de Jong E.M., Seyger M.M. (2010). Dithranol therapy in childhood psoriasis: Unjustifiably on the verge of falling into oblivion. Dermatology.

[55] Zvulunov A., Anisfeld A., Metzker A. (1994). Efficacy of short-contact therapy with dithranol in childhood psoriasis. Int. J. Dermatol..

[56] Oostveen A.M., Beulens C.A., van de Kerkhof P.C., de Jong E.M., Seyger M.M. (2014). The effectiveness and safety of short-contact dithranol therapy in paediatric psoriasis: A prospective comparison of regular day care and day care with telemedicine. Br. J. Dermatol..

[57] Menter M.A., Whiting D.A., McWilliams J. (1984). Resistant childhood psoriasis: An analysis of patients seen in a day-care center. Pediatr. Dermatol..

[58] Kortuem K.R., Davis M.D., Witman P.M., McEvoy M.T., Farmer S.A. (2010). Results of Goeckerman treatment for psoriasis in children: A 21-year retrospective review. Pediatr. Dermatol..

[59] Borska L., Andrys C., Krejsek J., Palicka V., Chmelarova M., Hamakova K., Kremlacek J., Fiala Z. (2014). Oxidative damage to nucleic acids and benzo(a)pyrene-7,8-diol-9,10-epoxide-DNA adducts and chromosomal aberration in children with psoriasis repeatedly exposed to crude coal tar ointment and UV radiation. Longev. OMAC.

[60] Smith S.H., Jayawickreme C., Rickard D.J., Nicodeme E., Bui T., Simmons C., Coquery C.M., Neil J., Pryor W.M., Mayhew D. (2017). Tapinarof Is a Natural AhR Agonist that Resolves Skin Inflammation in Mice and Humans. J. Investig. Dermatol. Symp. Proc..

[61] Smith S.H., McHale K., Creech K., Rickard D., Jayawickreme C., Wu D., Rastinejad F., Rubenstein D. (2020). Differential ligand binding distinguishes therapeutic from pathologic aryl hydrocarbon receptor (AhR) modulating agents: Implications for inflammatory skin disease. J. Investig. Dermatol..

[62] Bissonnette R., Bolduc C., Maari C., Nigen S., Webster J.M., Tang L. (2012). Efficacy and safety of topical WBI-1001 in patients with mild to moderate psoriasis: Results from a randomized double-blind placebo-controlled, phase II trial. J. Eur. Acad. Dermatol. Venereol..

[63] Paller A.S., Stein Gold L., Soung J., Tallman A.M., Rubenstein D.S., Gooderham M. (2021). Efficacy and patient-reported outcomes from a phase 2b, randomized clinical trial of tapinarof cream for the treatment of adolescents and adults with atopic dermatitis. J. Am. Acad. Dermatol..

[64] Walters I.B., Burack L.H., Coven T.R., Gilleaudeau P., Krueger J.G. (1999). Suberythemogenic narrow-band UVB is markedly more effective than conventional UVB in treatment of psoriasis vulgaris. J. Am. Acad. Dermatol..

[65] Ersoy-Evans S., Altaykan A., Sahin S., Kölemen F. (2008). Phototherapy in childhood. Pediatr. Dermatol..

[66] Jain V.K., Aggarwal K., Jain K., Bansal A. (2007). Narrow-band UV-B phototherapy in childhood psoriasis. Int. J. Dermatol..

[67] Bronckers I., Seyger M., West D.P., Lara-Corrales I., Tollefson M., Tom W.L., Hogeling M., Belazarian L., Zachariae C., Mahe E. (2017). Safety of Systemic Agents for the Treatment of Pediatric Psoriasis. JAMA Dermatol..

[68] Lara-Corrales I., Ramnarine S., Lansang P. (2013). Treatment of childhood psoriasis with phototherapy and photochemo-therapy. Clin. Med. Insights Pediatr..

[69] Kaur I., Dogra S., De D., Kanwar A.J. (2008). Systemic methotrexate treatment in childhood psoriasis: Further experience in 24 children from India. Pediatr. Dermatol..

[70] Warren R.B., Chalmers R.J., Griffiths C.E., Menter A. (2008). Methotrexate for psoriasis in the era of biological therapy. Clin. Exp. Dermatol..

[71] Herfarth H.H., Kappelman M.D., Long M.D., Isaacs K.L. (2016). Use of Methotrexate in the Treatment of Inflammatory Bowel Diseases. Inflamm. Bowel. Dis..

[72] Ergun T., Seckin Gencosmanoglu D., Alpsoy E., Bulbul-Baskan E., Saricam M.H., Salman A., Onsun N., Sarioz A. (2017). Efficacy, safety and drug survival of conventional agents in pediatric psoriasis: A multicenter, cohort study. J. Dermatol..

[73] van Geel M.J., Mul K., de Jager M.E., van de Kerkhof P.C., de Jong E.M., Seyger M.M. (2015). Systemic treatments in paediatric psoriasis: A systematic evidence-based update. J. Eur. Acad. Dermatol. Venereol..

[74] Rendon A., Schäkel K. (2019). Psoriasis Pathogenesis and Treatment. Int. J. Mol. Sci..

[75] Kiliç S.S., Hacimustafaoğlu M., Celebi S., Karadeniz A., Ildirim I. (2001). Low dose cyclosporin A treatment in generalized pustular psoriasis. Pediatr. Dermatol..

[76] (2015). Cyclosporine [Package Insert].

[77] Bulbul Baskan E., Yazici S., Tunali S., Saricaoglu H. (2016). Clinical experience with systemic cyclosporine A treatment in severe childhood psoriasis. J. Dermatolog. Treat..

[78] Dogra S., Mahajan R., Narang T., Handa S. (2017). Systemic cyclosporine treatment in severe childhood psoriasis: A retro-spective chart review. J. Dermatol. Treat..

[79] Lebwohl M. (2018). Psoriasis. Ann. Intern. Med..

[80] Rosińska D., Wolska H., Jablonska S., Konca I. (1988). Etretinate in severe psoriasis of children. Pediatr. Dermatol..

[81] Napolitano M., Megna M., Balato A., Ayala F., Lembo S., Villani A., Balato N. (2016). Systemic Treatment of Pediatric Psoriasis: A Review. J. Drugs Dermatol..

[82] Popadic S., Nikolic M. (2014). Pustular psoriasis in childhood and adolescence: A 20-year single-center experience. Pediatr. Dermatol..

[83] (2018). Absorica (Isotretinoin) [Package Insert].

[84] Halverstam C.P., Zeichner J., Lebwohl M. (2006). Lack of significant skeletal changes after long-term, low-dose retinoid therapy: Case report and review of the literature. J. Cutan Med. Surg..

[85] Marqueling A.L., Cordoro K.M. (2013). Systemic treatments for severe pediatric psoriasis: A practical approach. Dermatol. Clin..

[86] Mrowietz U., Barker J., Boehncke W.H., Iversen L., Kirby B., Naldi L., Reich K., Tanew A., van de Kerhok P.C.M., Warren R.M. (2018). Clinical use of dimethyl fumarate in moderate-to-severe plaque-type psoriasis: A European expert consensus. J. Eur. Acad. Dermatol. Venereol..

[87] Steinz K., Gerdes S., Domm S., Mrowietz U. (2014). Systemic treatment with fumaric acid esters in six paediatric patients with psoriasis in a psoriasis centre. Dermatology.

[88] Paller A.S., Siegfried E.C., Langley R.G., Gottlieb A.B., Pariser D., Landells I., Herbert A.A., Eichenfield L.F., Patel V., Creamer K. (2008). Etanercept treatment for children and adolescents with plaque psoriasis. N. Engl. J. Med..

[89] Aslam N., Saleem H., Murtazaliev S., Quazi S.J., Khan S. (2020). FDA Approved Biologics: Can Etanercept and Ustekinumab be Considered a First-Line Systemic Therapy for Pediatric/Adolescents in Moderate to Severe Psoriasis? A Systematic Review. Cureus.

[90] Langley R.G., Paller A.S., Hebert A.A., Creamer K., Weng H.H., Jahreis A., Globe D., Patel V., Orlow S.J. (2011). Patient-reported outcomes in pediatric patients with psoriasis undergoing etanercept treatment: 12-week results from a phase III randomized controlled trial. J. Am. Acad. Dermatol..

[91] Fabrizi G., Guerriero C., Pagliarello C. (2007). Etanercept in infants: Suberythrodermic, recalcitrant psoriasis in a 22 month-old child successfully treated with etanercept. Eur. J. Dermatol..

[92] Paller A.S., Siegfried E.C., Eichenfield L.F., Pariser D., Langley R.G., Creamer K., Kricorian G. (2010). Long-term etanercept in pediatric patients with plaque psoriasis. J. Am. Acad. Dermatol..

[93] Paller A.S., Siegfried E.C., Pariser D.M., Rice K.C., Trivedi M., Iles J., Collier D.H., Kricorian G., Langley R.G. (2016). Long-term safety and efficacy of etanercept in children and adolescents with plaque psoriasis. J. Am. Acad. Dermatol..

[94] Papp K., Thaçi D., Marcoux D., Weibel L., Philipp S., Ghislain P.D., Landells I., Hoeger P., Kotkin C., Unnebrink K. (2017). Efficacy and safety of adalimumab every other week versus methotrexate once weekly in children and adolescents with severe chronic plaque psoriasis: A randomised, double-blind, phase 3 trial. Lancet.

[95] Xie X., Wang Y., Yao S., Xia Y., Luo H., Li L., Lu C. (2021). Biologics recommendations for patients with psoriasis: A critical appraisal of clinical practice guidelines for psoriasis. J. Dermatolog Treat..

[96] Saeki H., Terui T., Morita A., Sano S., Imafuku S., Asahina A., Komine M., Etoh T., Igarashi A., Torii H. (2020). Japanese guidance for use of biologics for pso-riasis (the 2019 version). J. Dermatol..

[97] Landells I., Marano C., Hsu M.C., Li S., Zhu Y., Eichenfield L.F., Hoeger P.H., Menter A., Paller A.S., Taieb A. (2015). Ustekinumab in adolescent patients age 12 to 17 years with moderate-to-severe plaque psoriasis: Results of the randomized phase 3 CADMUS study. J. Am. Acad. Dermatol..

[98] Klufas D.M., Wald J.M., Strober B.E. (2016). Treatment of Moderate to Severe Pediatric Psoriasis: A Retrospective Case Series. Pediatr. Dermatol..

[99] Lim H., Lee S.H., Lee H.T., Lee J.U., Son J.Y., Shin W., Heo Y.-S. (2018). Structural Biology of the TNFα Antagonists Used in the Treatment of Rheumatoid Arthritis. Int. J. Mol. Sci..

[100] Pinson R., Sotoodian B., Fiorillo L. (2016). Psoriasis in children. Psoriasis.

[101] Bodemer C., Kaszuba A., Kingo K., Tsianakas A., Morita A., Rivas E., Papanastasiou P., Keefe D., Patekar M., Charef P. (2021). Secukinumab demonstrates high effi-cacy and a favourable safety profile in paediatric patients with severe chronic plaque psoriasis: 52-week results from a Phase 3 double-blind randomized, controlled trial. J. Eur. Acad. Dermatol. Venereol..

[102] Paller A.S., Seyger M., Alejandro Magariños G., Bagel J., Pinter A., Cather J., Keller S., Capriles C.R., Lima R.G., Gallo G. (2020). Efficacy and safety of ixeki-zumab in a phase III, randomized, double-blind, placebo-controlled study in paediatric patients with moderate-to-severe plaque psoriasis (IXORA-PEDS). Br. J. Dermatol..

[103] Blauvelt A., Papp K.A., Griffiths C.E., Randazzo B., Wasfi Y., Shen Y.K., Li S., Kimball A.B. (2017). Efficacy and safety of guselkumab, an anti-interleukin-23 monoclonal antibody, compared with adalimumab for the continuous treatment of patients with moderate to severe psoriasis: Results from the phase III, double-blinded, placebo- and active comparator-controlled VOYAGE 1 trial. J. Am. Acad. Dermatol..

[104] Papp K.A., Blauvelt A., Bukhalo M., Gooderham M., Krueger J.G., Lacour J.P., Menter A., Philipp S., Sofen H., Tyring S. (2017). Risankizumab versus Ustekinumab for Moderate-to-Severe Plaque Psoriasis. N. Engl. J. Med..

[105] Papp K.A., Menter M.A., Abe M., Elewski B., Feldman S.R., Gottlieb A.B., Langley R., Luger T., Thaci D., Buonanno M. (2015). Tofacitinib, an oral Janus kinase inhibitor, for the treatment of chronic plaque psoriasis: Results from two randomized, placebo-controlled, phase III trials. Br. J. Dermatol..

[106] Bachelez H., van de Kerkhof P.C., Strohal R., Kubanov A., Valenzuela F., Lee J.H., Yakusevich V., Chimenti S., Papacharalambous J., Proulx J. (2015). Tofacitinib versus etanercept or placebo in moderate-to-severe chronic plaque psoriasis: A phase 3 randomised non-inferiority trial. Lancet.

[107] AlMutairi N., Nour T. (2020). Tofacitinib in Pediatric Psoriasis: An Open-Label Trial to Study Its Safety and Efficacy in Children. Dermatology.

